# Assessment of subclinical left ventricular myocardial systolic dysfunction in type 2 diabetes mellitus patients with or without hypertension by global and segmental myocardial work

**DOI:** 10.1186/s13098-023-01180-0

**Published:** 2023-10-13

**Authors:** Guang-An Li, Jun Huang, Xiao Sheng, Li Fan

**Affiliations:** 1 Department of Echocardiography, The Affiliated Changzhou Second People’s Hospital with Nanjing Medical University, Changzhou, 213003 China; 2 Department of Endocrinology, The Affiliated Changzhou Second People’s Hospital with Nanjing Medical University, Changzhou, 213003 China

**Keywords:** Subclinical left ventricular myocardial systolic dysfunction, Myocardial work, Speckle tracking echocardiography, Type 2 diabetes mellitus, Hypertension

## Abstract

**Objective:**

The research was aimed to evaluate the subclinical left ventricular (LV) myocardial systolic dysfunction in T2DM patients with or without hypertension (HT) by global and segmental myocardial work (MW).

**Methods:**

A total of 120 T2DM patients (including 60 T2DM patients with HT) and 70 sex- and age- matched normal controls were included. The global and segmental variables of work index (WI), constrictive work (CW), waste work (WW), work efficiency (WE), and CW/WW were analysed by non-invasive pressure-strain loop. Receiver operating characteristic (ROC) analysis was performed for detection the subclinical LV systolic dysfunction in T2DM patients with and without HT.

**Results:**

The global work index (GWI), global CW (GCW), global WE (GWE), and GCW/global WW (GWW) of T2DM and T2DM patients with HT were significantly lower than normal controls (p < 0.05). The WI, CW, WE, and CW/WW of the LV anterior wall in T2DM and T2DM patients with HT were significantly lower when compared with those of the normal controls (p < 0.05). ROC analysis showed that the value of area under the curve (AUC) in combined GWI, GCW, GWE, and GCW/GWW was significantly higher than the AUCs of the individual indices (p < 0.05).

**Conclusions:**

MW can non-invasively and accurately evaluate subclinical global and segmental LV myocardial systolic dysfunction in T2DM patients with and without HT. Regulating total cholesterol levels and controlling blood pressure in T2DM patients with and without HT might reduce the impairment of LV myocardial systolic function.

## Introduction

With the growth of the population and increased longevity, non-infectious chronic diseases (NCDs) are still the main causes of poor health worldwide. In a report released by the World Health Organization in 2022 [1], as one of the most common NCDs, type 2 diabetes mellitus (T2DM) has shown an increasing trend in both incidence and mortality over the last 20 years. Cardiovascular complications are viewed as dangerous complications of T2DM culminating in death [2]. Although ischaemic events dominate cardiovascular complications, in the absence of myocardial ischaemia and conditions of hypertension, diabetic cardiomyopathy also carries an increased risk of progression to heart failure [3]. In previous studies in which left ventricular (LV) global longitudinal strain (GLS) was measured using speckle-tracking echocardiography (STE), patients with T2DM who developed myocardial dysfunction could be identified even when LV ejection fraction (LVEF) was preserved [4, 5]. The acquisition of LV global radial, circumferential, and longitudinal strain by cardiovascular magnetic resonance (CMR) tissue tracking also enables the evaluation of myocardial systolic function in T2DM patients [6]. Due to the high cost and time-consuming nature of CMR, as well as LVGLS is excessively load dependent. Therefore, their ability to serve as accurate, widely used indicators of myocardial systolic function is limited [7, 8].

Myocardial work (MW) estimation has been validated as a novel, non-invasive method for constructing the LV pressure-strain loop (PSL) area, with good correlation with directly measured MW, and it is able to reflect the systolic and metabolic capacity of the myocardium [9, 10]. Previously, several studies have shown that the global myocardial work index (GWI) is significantly impaired in T2DM patients compared with normal controls [5, 11]. Nevertheless, subclinical LV myocardial systolic dysfunction has not been evaluated in T2DM patients with hypertension (HT), and whether there is LV segmental myocardial impairment in T2DM patients is unknown. Therefore, the aims of the present study were to (1) evaluate subclinical global and segmental LV myocardial systolic dysfunction by MW in T2DM patients with and without HT, (2) evaluate the influences of patients' clinical indices on myocardial systolic function, and (3) evaluate the sensitivity and specificity of MW in evaluating subclinical LV myocardial systolic dysfunction in T2DM patients with and without hypertension (HT).

## Methods

### Study population

**Fig. 1:**
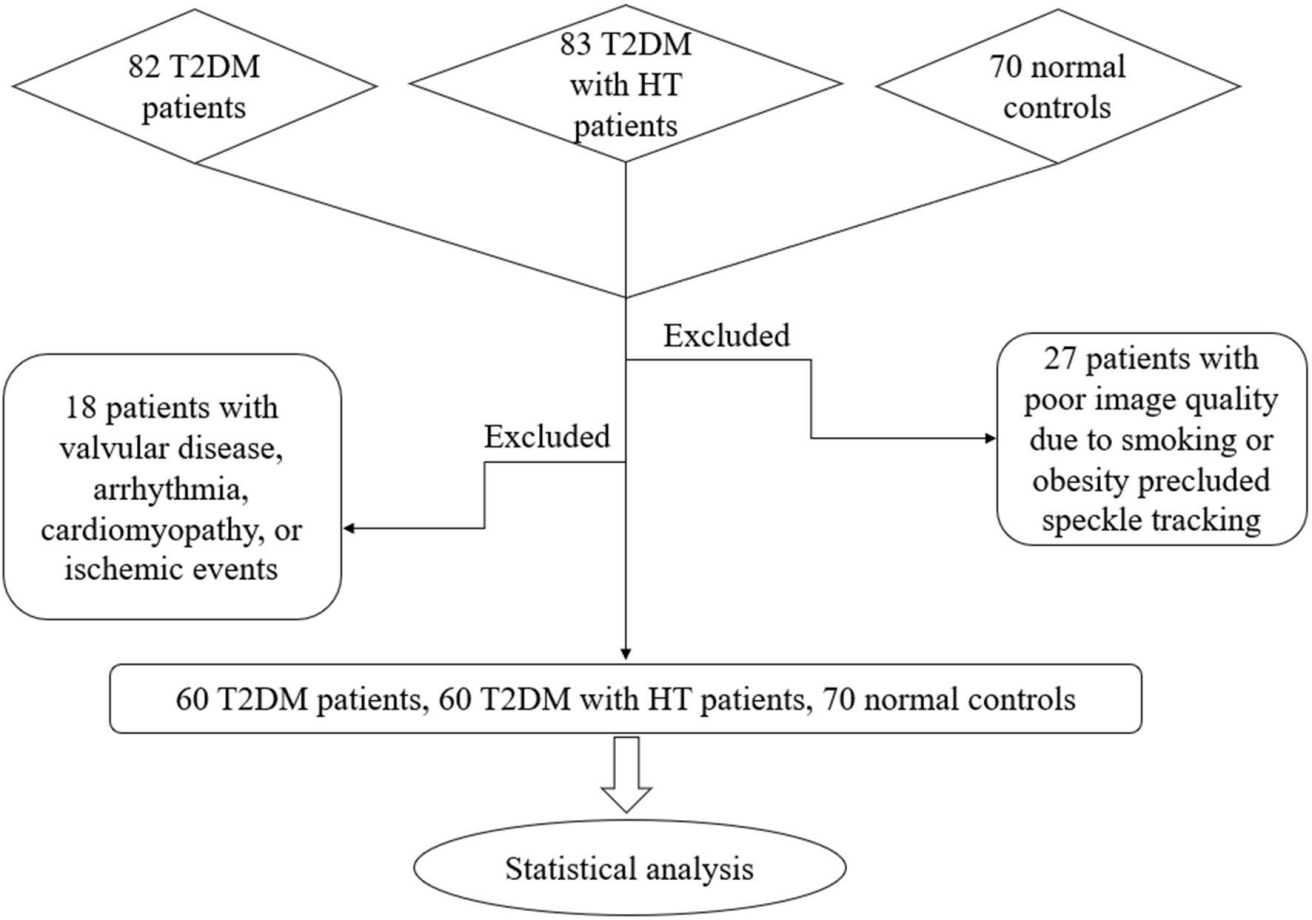
Flow chart of participant screening for the study cohort

The study population included 165 T2DM patients who had already been diagnosed or were diagnosed during hospitalization and 83 T2DM patients who were simultaneously diagnosed with HT or had a history of HT. The diagnostic criteria for T2DM conformed to the latest guidelines [12]: A1C ≥ 6.5%, fasting plasma glucose (FPG) ≥ 126 mg/dl (7.0 mmol/L), 2-h plasma glucose ≥ 200 mg/dl (11.1 mmol/L) during an oral glucose tolerance test (OGTT) or random plasma glucose ≥ 200 mg/dl (11.1 mmol/L) + typical hyperglycaemia symptoms. The diagnosis of hypertension followed the standards of the European Society of Cardiology [13]: systolic blood pressure (SBP) ≥ 140 mmHg and/or diastolic blood pressure (DBP) ≥ 90 mmHg on two separate occasions or regular antihypertensive treatment. According to their clinical status, T2DM patients with (1) valvular disease, (2) arrhythmia, (3) cardiomyopathy, (4) previous or current ischemic events, (5) poor image quality, were excluded. Furthermore, 70 sex- and age-matched normal individuals were recruited as normal controls (Fig. 1). The study adhered to the guidelines of the Declaration of Helsinki. Approval was obtained from the human research and ethics committee of Changzhou Second People's Hospital Affiliated to Nanjing Medical University, and all patients provided complete informed consent.

### Conventional echocardiography

An ultrasound machine (Vivid E9; GE Vingmed Ultrasound, Horten, Norway) was used by senior sonographers to perform standard 2-dimensional transthoracic echocardiography. 40–60 frames of standard images in three cardiac cycles at the apical two-, three- and four- chamber views were collected, and the electrocardiogram was connected while images were collected. Proprietary software (EchoPAC 203; GE Vingmed Ultrasound) was used to store images and perform offline analysis.

Left atrium diameter (LAd), interventricular septum thickness diameters (IVSd), LV diameter (LVd) and LV posterior wall thickness diameters (LVPWd) were measured from the parasternal long-axis views in the end-diastole period [14].Maximal left atrial volume was measured from apical four- and two-chamber views, and correction of body surface area (BSA) differences between individuals was performed using the left atrial volume (LAV) index. Peak mitral valve medial flow velocity (E) and lateral annulus flow velocity (e′) in the early diastolic period were measured using the Doppler technique in the apical four-chamber view [15]. At the same time, the peak late diastolic annular velocity (A) was measured.

The displacement waveform of the mitral annulus during systole was measured from M-mode echocardiography in apical four-chamber views, with the distance between the peak point and the nadir as the magnitude of the mitral annular plane systolic excursion (MAPSE) [16]. LV end-systolic volume (LVESV), LV end-diastolic volume (LVEDV) and LVEF were measured by the biplane Simpson method [14]. Correction of BSA differences between individuals was executed using the LVESV and LVEDV indexes.

### Myocardial work

The image analysis in this study was usually performed offline by two senior sonographers using EchoPAC 203. The regions of interest were automatically delineated in the acquired standard apical four-, two- and three-chamber views by STE, and if necessary, the endocardial borders were manually adjusted to better track myocardial motion. LV GLS was derived based on averaging the peak strain of 17 segments from three apical views. The ejection period was defined according to the open cut points of the mitral and aortic valves. LV PSL was constructed by non-invasive measurement of LV systolic pressure combined with LV GLS.

The area of LV PSL is equal to the LV GWI. During systole, the work done to promote the shortening of the myocardium is constructive work (CW), and myocardial work performed during elongation is waste work (WW). At isovolumetric diastole, the work contributing to myocardial lengthening is the CW, and the work of myocardial shortening is defined as the WW.

$$ {\text{Work}}\,{\text{efficiency}}\left( {WE} \right) = CW/(CW + WW) \times 100\% . $$

The basal, mid, and apical sections of the myocardium were confluent to generate six segments (septal, anteroseptal, inferior, lateral, posterior, and anterior) to evaluate segmental differences in MW.

### Clinical parameters

All patients and normal controls underwent blood collection on the day of image collection, and blood biochemical parameters were analysed by the Department of Laboratory Medicine. The glycated haemoglobin A1c (HbA1c) of patients was obtained through HLC-723 G11 (Tosoh Bioscience) and its corresponding kit. The total cholesterol (TC), triglycerides (TG), high density lipoprotein cholesterol (HDL-C), low density lipoprotein cholesterol (LDL-C), fasting plasma glucose (FPG), blood urea nitrogen (BUN), and serum creatinine (SCR) concentrations of patients were obtained through Roche Cobas 8000 and these corresponding kits.

### Statistical analysis

SPSS statistical software, version 23.0 (SPSS Inc., Chicago, USA), was used for the statistical analysis of all data. Statistical significance was indicated by a p value of < 0.05. The Shapiro‒Wilk test was used to test whether the data conformed to normal distribution. Continuous variable data are presented as the mean ± SD when normally distributed and the median (interquartile range) when not normally distributed. Absolute values and percentiles were used to represent categorical data. When comparing normal controls, T2DM patients, and T2DM patients with HT, one-way analysis of variance (ANOVA), the Kruskal‒Wallis test, or the x^2^ test were applied. Univariate regression analysis was first used to evaluate the relationship between traditional echocardiographic or clinical parametersand global and segmental myocardial work parameters. Each relevant parameter with a p value of < 0.20 was included in the multivariate regression analysis. The parameters of MW in the normal controls were defined as normal, and the corresponding indexes in T2DM patients with and without HT were defined as abnormal. The area under the receiver operating characteristic (ROC) curve (AUC) was used to evaluate the sensitivity and specificity of MW in evaluating subclinical LV myocardial systolic dysfunction in T2DM patients with and without HT. Reproducibility was tested by intra- and intergroup correlation coefficient analysis.

## Results

### Study population

The clinical parameters of the study cohort are shown in Table 1. 27 patients with poor image quality due to smoking or obesity that precluded speckle tracking and 18 patients with valvular disease, arrhythmia, cardiomyopathy, or ischaemic events were excluded. After recruitment and screening, a total of 190 individuals were included in the study: 60 T2DM patients (mean age, 48.58 ± 11.69 years); 60 T2DM patients with HT (mean age, 52.86 ± 13.13 years); and 70 sex- and age- matched normal individuals (mean age, 48.53 ± 9.68 years). There was no significant difference in age or sex among the three groups (p > 0.05). However, the body mass index (BMI) and body surface area (BSA) of T2DM patients with and without HT were remarkably higher than those of the normal controls (p < 0.05). The SBP and DBP in T2DM patients with HT were significantly higher than those in the other two groups (p < 0.05).

**Table 1:** Comparison of clinical parameters among the normal controls, T2DM, and T2DM with HT groups

**Clinical parameters**	Normal controls (n = 70)	T2DM (n = 60)	T2DM with dyslipidemia (n = 60)	p value
Age, year	48.53 ± 9.68	48.58 ± 11.69	52.86 ± 13.13	0.074
Male, n (%)	31 (44%)	36 (60%)	35 (58%)	0.137
BMI, kg/m^2^	24.00 ± 3.31	25.98 ± 3.52*	26.63 ± 4.02*	< 0.001
BSA, m^2^	1.69 ± 0.20	1.79 ± 0.21*	1.80 ± 0.21*	0.005
SBP, mmHg	126.17 ± 9.48	124.98 ± 11.97	135.41 ± 16.38*^#^	< 0.001
DBP, mmHg	80.03 ± 7.28	79.17 ± 9.56	85.64 ± 9.97*^#^	< 0.001
HbA1c, %	5.46 ± 0.38	9.78 ± 2.07*	8.62 ± 2.08*^#^	< 0.001
TC, mmol/L	4.56 ± 0.89	4.36 ± 0.91	4.57 ± 1.03	0.237
TG, mmol/L	1.36 (1.22,1.78)	1.78 (1.61,2.25)*	1.94 (1.93,2.75)*	< 0.001
HDL-C, mmol/L	1.28 ± 0.33	1.07 ± 0.26*	1.00 ± 0.26*	< 0.001
LDL-C, mmol/L	2.67 ± 0.75	2.69 ± 0.74	2.76 ± 0.89	0.448
FPG, mmol/L	4.95 (4.82, 5.14)	10.68 (10.58, 13.36) *	9.10 (9.62, 12.96)*	< 0.001
BUN, mmol/L	4.70 (4.23, 5.32)	5.45 (5.02, 5.89)	5.50 (5.60, 7.24)*	0.008
SCR, μmol/L	61.80 (60.35, 70.58)	58.05 (54.90, 63.91)	60.55 (61.05, 91.35)^#^	0.026
Medication				
ACEI/ARB, n (%)	–	–	24 (40)	
Calcium channel blocker, n (%)	–	–	29 (48.3)	
B-blocker, n (%)	–	–	4 (6.7)	
Diuretic, n (%)	–	–	6 (10)	
SGLT-2 inhibitor, n (%)	–	8 (13.3)	24 (60)	
Metformin, n (%)	–	36 (60)	32 (53.3)	
Insulin, n (%)	–	34 (56.7)	34 (56.7)	

*BMI* body mass index, *BSA* body surface area, *SBP* systolic blood pressure, *DBP* diastolic blood pressure, *HbA1c* glycosylated hemoglobin A1c, *TC* total cholesterol, *TG* triglyceride, *HDL-C* high-density lipoprotein cholesterol, *LDL-C* low-density lipoprotein cholesterol, FPG fasting plasma glucose, *BUN* blood urea nitrogen, *SCR* serum creatinine

Data are expressed as Mean ± SD, number (percentage), or median (interquartile range)

*p < .05 vs normal controls

^#^p < .05 vs T2DM

### Conventional echocardiographic parameters

Traditional echocardiographic parameters of the study cohort are shown in Table 2. The LAd (p = 0.011), LVPWd (p < 0.001) and IVSd (p < 0.001) were significantly increased in T2DM patients with HT compared with patients with T2DM alone and normal controls. The MAPSE (p < 0.05), E/A (p < 0.001) and E/e′ (p < 0.05) of T2DM patients with HT were significantly lower than those of T2DM patients and normal controls. The LVEDV index (p < 0.001) and LVESV index (p = 0.025) of T2DM patients and T2DM patients with HT were significantly lower than those of normal controls. However, the LVEF of the three groups remained within the normal ranges.

**Table 2:** Comparison of traditional echocardiographic parameters among the normal controls, T2DM, and T2DM with HT groups

Echocardiographic parameters	Normal controls (n = 70)	T2DM (n = 60)	T2DM with HT (n = 60)	p value
LA diameter, mm	34.30 ± 2.76	35.33 ± 2.70	36.33 ± 3.40*^#^	0.011
LAV index, ml/m^2^	30.88 ± 7.58	29.63 ± 6.40	30.52 ± 7.65	0.608
IVS diameter, mm	9.23 ± 0.82	8.99 ± 0.95	9.76 ± 0.83*^#^	< 0.001
PW diameter, mm	8.97 ± 0.82	8.64 ± 0.89	9.50 ± 1.06*^#^	< 0.001
LV diameter, mm	45.75 ± 2.74	46.46 ± 3.70	46.66 ± 2.99	0.669
LVEDV index, ml/m^2^	49.70 ± 9.17	44.77 ± 7.90*	43.35 ± 9.69*	< 0.001
LVESV index, ml/m^2^	18.75 ± 4.07	17.20 ± 3.64*	16.96 ± 4.49*	0.025
LVEF, %	65.44 ± 2.92	61.78 ± 3.26	61.15 ± 3.11	0.061
MAPSE, mm	14.11 ± 2.45	14.52 ± 1.53	13.43 ± 2.16*^#^	0.023
E, m/s	0.82 ± 0.12	0.76 ± 0.14*	0.75 ± 0.15*	0.014
A, m/s	0.68 ± 0.15	0.70 ± 0.17	0.80 ± 0.20*	< 0.001
E/A	1.24 (1.16,1.37)	1.06 (1.05,1.24)	0.92 (0.90,1.12)*^#^	< 0.001
e′, m/s	0.11 ± 0.02	0.10 ± 0.02*	0.09 ± 0.02*^#^	< 0.001
E/e′	7.82 ± 1.48	8.25 ± 1.65	8.91 ± 2.44*^#^	0.001
LVGLS, %	− 20.55 ± 1.90	− 18.87 ± 2.04*	− 17.90 ± 1.94*^#^	< 0.001

*LA* left atrial, *LAV* left atrial volume, *IVS* interventricular septum, *PW* posterior wall, *LV* left ventricular, *LVEDV* LV end-diastolic volume, *LVESV* LV end-systolic volume, *LVEF* left ventricular ejection fraction, *MAPSE* mitral annular plane systolic excursion, *LVGLS* LV global longitudinal strain

Data are expressed as Mean ± SD, or median (interquartile range)

*p < .05 vs normal controls

^#^p < .05 vs T2DM

### Global myocardial work parameters

**Fig. 2:**
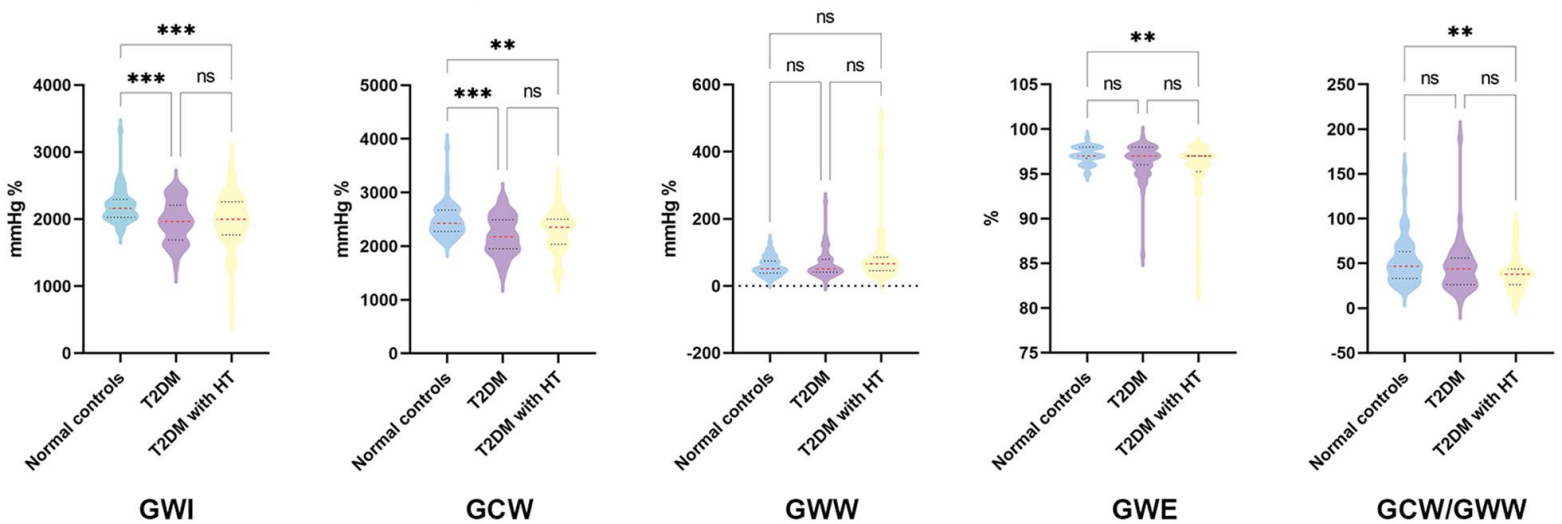
Differences in global MW parameters among normal controls, T2DM patients and T2DM patients with HT. **: significant differences were found between groups (p < 0.05). ***: p < 0. 001.ns: no significant differences were found between groups (p > 0.05)

The global MW parameters of the study cohort are shown in Table 3 and Fig. 2. Significant reductions in GWI and GCW were observed in T2DM patients and T2DM patients with HT compared with normal controls (p < 0.001). In addition, from normal controls to T2DM patients with HT, GWI and GCW showed a decreasing trend first and then an increasing trend (p_trend_ < 0.05). GWE (p = 0.002) and GCW/GWW (p = 0.004) appeared significantly lower in T2DM patients with HT than in normal controls, and both showed a decreasing trend (p_trend_ < 0.05). Although the divergences in GWW among the three groups were not significant (p = 0.064), the overall value showed an increasing trend (p_trend_ = 0.003).

**Table 3:** Comparison of LV global MW parameters among the normal controls, T2DM, and T2DM with HT groups

**Global MW**	Normal controls (n = 70)	T2DM (n = 60)	T2DM with HT (n = 60)	p value	p _trend_
GWI, mmHg%	2186.87 ± 234.74	1965.82 ± 301.13*	1966.95 ± 398.90*	< 0.001	< 0.001
GCW, mmHg%	2472.11 ± 295.81	2204.67 ± 350.34*	2276.36 ± 380.65*	< 0.001	0.002
GWW, mmHg%	51.00 (51.86,64.14)	50.50 (53.96,74.21)	67.00 (64.79,110.39)	0.064	0.003
GWE, %	97 (97,97)	97 (96,97)	97 (95,97)*	0.002	< 0.001
GCW/GWW	46.82 (45.68,57.57)	43.67 (38.09,51.92)	37.72 (33.17,43.70)*	0.004	0.002

*GWI* global myocardial work index, *GCW* global constructive work, *GWW* global waste work, *GWE* global work efficiency

*p < .05 vs normal controls

### Correlation and exposure factors

As shown in Table 4. GCW showed certain correlations with sex (p = 0.021), SBP (p < 0.001) and MAPSE (p < 0.001) in T2DM patients with and without HT.

**Table 4:** Relationship between GCW and different clinical and echocardiographic parameters in T2DM patients with and without HT

Variables	Univariable		Multivariable	
	r	p	β	p
Sex	0.21	0.021	0.11	0.122
Age, year	− 0.13	0.149	0.08	0.305
BMI, kg/m2	0.10	0.282	N/A	N/A
HbA1c, %	− 0.01	0.932	N/A	N/A
TC, mmol/L	0.14	0.136	− 0.15	0.040
FPG, mmol/L	0.11	0.241	N/A	N/A
BUN, mmol/L	− 0.17	0.081	− 0.12	0.085
SBP, mmHg	0.66	< 0.001	0.70	< 0.001
LVEF, %	− 0.01	0.908	N/A	N/A
MAPSE, mm	0.32	< 0.001	0.33	< 0.001
				R^*2*^ = 0.59

*BMI* body mass index, *HbA1c* glycosylated hemoglobin type A1c, *TC* total cholesterol, *FPG* fasting plasma glucose, *BUN* blood urea nitrogen, *SBP* systolic blood pressure, *LVEF* left ventricular ejection fraction, *MAPSE* mitral annular plane systolic excursion

Multivariate regression analysis was applied to appraise the independent effects of the above indicators on GCW. TC (p = 0.040), SBP (p < 0.001), and MAPSE (p < 0.001) were were independently associated with GCW in T2DM patients with and without HT.

### Construction and analysis of an ROC curve

**Fig. 3:**
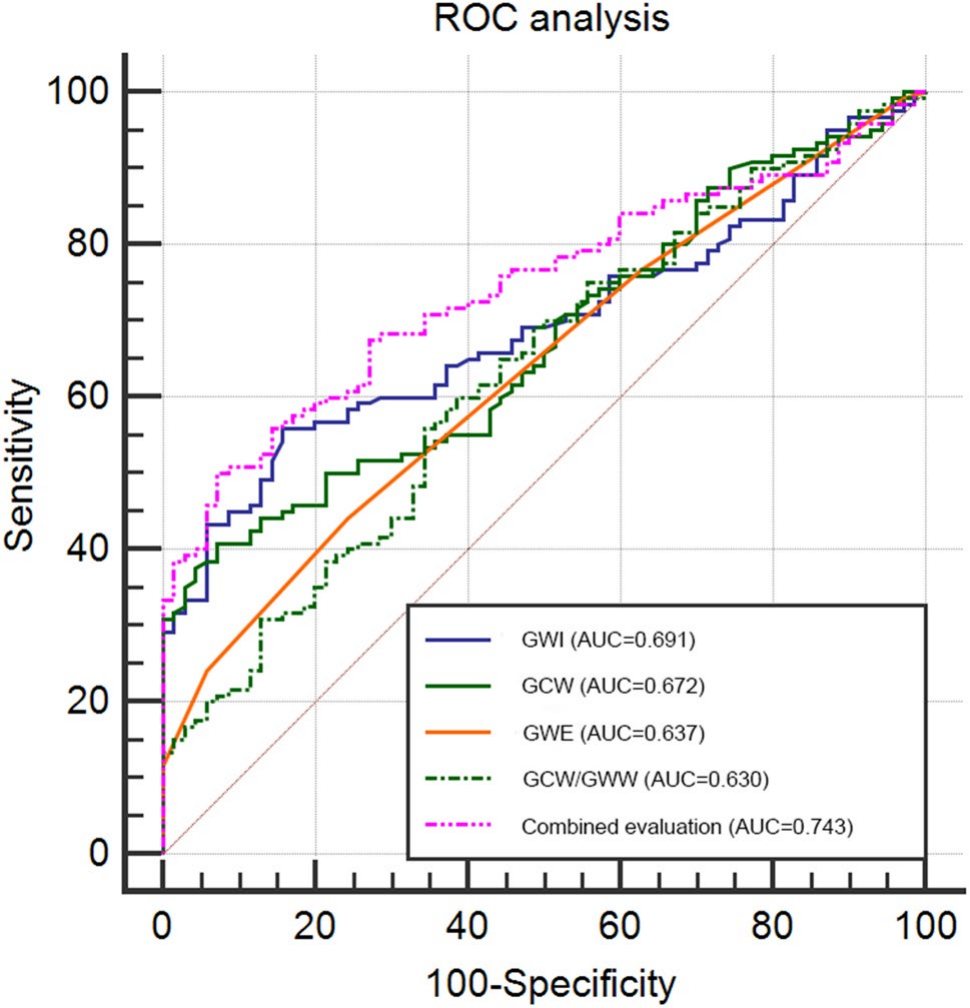
ROC analysis was used to determine the AUC, sensitivity and specificity of MW parameters to evaluate subclinical LV myocardial systolic function in T2DM patients

An ROC curve was constructed to analyse whether MW could accurately evaluate subclinical LV myocardial dysfunction in T2DM patients with and without HT (Table 5 and Fig. 3). The sensitivity and specificity of the GWI were 55.83% and 84.29%, respectively, with an area under the curve (AUC) of 0.691. The sensitivity and specificity of GCW were 40.83% and 92.86%, respectively, with an AUC of 0.672. The sensitivity and specificity of GWE were 44.17% and 75.71%, respectively, with an AUC of 0.637. The sensitivity and specificity of GCW/GWW were 55.83% and 65.71%, respectively, with an AUC of 0.630. The sensitivity and specificity of combined GWI, GCW, GWE, and GCW/GWW were 50.00% and 92.86%, respectively, with an AUC of 0.743, and the value of AUC in combined these variables was significantly higher than the AUCs of the individual indices (p < 0.05).

**Table 5:** ROC analysis of MW parameters in T2DM patients with and without HT

ROC	GWI	GCW	GWE	GCW/GWW	Combined evaluation
Sensitivity, %	55.83	40.83	44.17	55.83	50.00
Specificity, %	84.29	92.86	75.71	65.71	92.86
AUC (95%CI)	0.691 (0.621 to 0.756)*	0.672 (0.601 to 0.738)*	0.637 (0.564 to 0.705)*	0.630 (0.557 to 0.699)*	0.743 (0.675 to 0.804)
Associated criterion	2003.00	2128.00	96.00	40.96	0.74
p	< 0.001	< 0.001	< 0.001	0.002	< 0.001

*: Significant difference (p < 0.05) compared with combined evaluation

### Intra- and interobserver variability analysis

The intraclass correlation coefficients (ICCs) for GWI, GCW, GWW, GWE and GCW/GWW were 0.96 (95% CI: 0.90–0.98, p < 0.001), 0.94 (95% CI: 0.85–0.98, p < 0.001), 0.94 (95% CI: 0.85–0.98, p < 0.001), 0.89 (95% CI: 0.75–0.96, p < 0.001) and 0.88 (95% CI: 0.73–0.95, p < 0.001), respectively. The interobserver ICCs were 0.94 (95% CI: 0.86–0.98, p < 0.001) for GWI, 0.91 (95% CI: 0.78–0.96, p < 0.001) for GCW, 0.88 (95% CI: 0.73–0.95, p < 0.001) for GWW, 0.86 (95% CI: 0.69–0.94, p < 0.001) for GWE and 0.86 (95% CI: 0.69–0.94, p < 0.001) for GCW/GWW.

### Segmental MW parameters

**Fig. 4:**
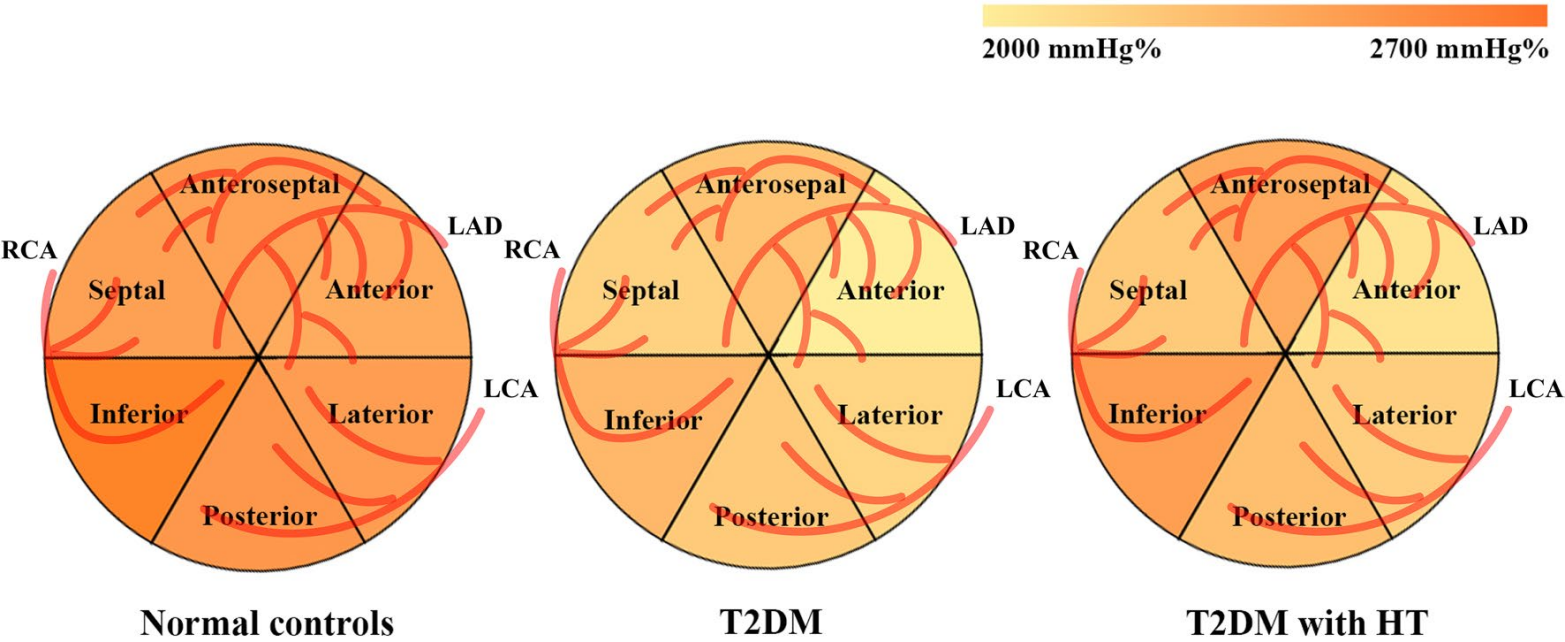
Difference in segmental CW between normal controls, T2DM patients, and T2DM patients with HT. *LAD* left anterior descending coronary artery, *LCA* left circumflex artery, *RCA* right coronary artery

The segmental MW parameters of the study cohort are shown in Table 6 and Fig. 4. There were no significant differences in the myocardial work index (MWI) (p = 0.331), CW (p = 0.100), WW (p = 0.114) or CW/WW (p = 0.110) of the anteroseptal wall among normal controls, T2DM patients and T2DM patients with HT. Differences of WE in anteroseptal wall appeared milder (p = 0.048). In contrast, the MWI (p = 0.010), CW (p < 0.001), WW (p = 0.005), WE (p < 0.001) and CW/WW (p < 0.001) of the anterior wall showed significant differences among the three groups. In addition, the MWI and CW of the inferior, posterior, lateral, and septal segment showed significant differences among the three groups (p < 0.05).

**Table 6:** Comparison of LV segmental MW parameters among the normal controls, T2DM, and T2DM with HT groups

LV segment	MWI (mmHg%)			p value	CW (mmHg%)			p value	WW (mmHg%)			p value
	Normal controls (*n* = 70)	T2DM (*n* = 60)	T2DM with HT (*n* = 60)		Normal controls (*n* = 70)	T2DM (*n* = 60)	T2DM with HT (*n* = 60)		Normal controls (*n* = 70)	T2DM (*n* = 60)	T2DM with HT (*n* = 60)	
Inferior	2392.44 ± 322.01	2207.60 ± 423.64*	2119.74 ± 456.20*	< 0.001	2665.99 ± 389.76	2321.49 ± 417.59*	2443.12 ± 495.12*	< 0.001	54.50 (54.24,75.75)	46.83 (50.47,78.00)	51.33 (49.95,95.08)	0.726
Posterior	2147.69 ± 297.11	2041.64 ± 473.71	1975.31 ± 465.65*	0.033	2481.51 ± 382.65	2231.41 ± 456.34*	2286.29 ± 429.56*	0.002	67.33 (64.70,86.70)	66.00 (64.38,117.40)	82.33 (76.14,133.59)	0.598
Lateral	2137.70 ± 252.11	2046.84 ± 423.32	1905.41 ± 475.06*	0.005	2445.94 ± 293.14	2179.14 ± 420.82*	2204.69 ± 410.57*	< 0.001	51.33 (48.56,66.63)	49.00 (47.64,70.36)	60.00 (60.15,156.46)	0.163
Anterior	2117.25 ± 377.90	2021.17 ± 492.54	1880.75 ± 471.06*	0.010	2380.10 ± 450.40	2045.19 ± 443.85*	2107.92 ± 509.18*	< 0.001	41.00 (39.92,56.97)	40.83 (42.74,68.44)	69.00 (60.92,90.42)*^#^	0.005
Anteroseptal	2126.92 ± 309.43	2066.06 ± 472.11	2018.45 ± 504.12	0.331	2426.21 ± 371.00	2261.62 ± 478.87	2387.27 ± 515.83	0.100	32.00 (35.90,53.16)	39.00 (38.25,83.04)	46.67 (56.89,99.75)	0.114
Septal	2198.99 ± 262.80	2048.47 ± 331.90*	1902.02 ± 448.96*^#^	< 0.001	2433.15 ± 308.60	2189.01 ± 382.66*	2228.82 ± 454.86*	< 0.001	49.50 (47.22,66.33)	45.67 (45.42,64.36)	45.33 (47.01,124.91)	0.991

LV segment	WE (%)			p value	CW/WW			p value
	Normal controls (*n* = 70)	T2DM (*n* = 60)	T2DM with HT (*n* = 60)		Normal controls (*n* = 70)	T2DM (*n* = 60)	T2DM with HT (*n* = 60)	
Inferior	97.33 (96.52,97.33)	97.33 (96.07,97.20)	97.33 (95.64,97.20)	0.955	47.10 (56.08,95.83)	51.34 (46.86,128.56)	47.58 (56.02,155.39)	0.908
Posterior	97.00 (96.06,96.92)	96.83 (94.18,96.77)	96.00 (93.87,96.23)	0.287	36.70 (39.63,68.84)	36.96 (37.35,67.46)	28.37 (28.76,75.11)	0.216
Lateral	97.33 (96.75,97.46)	97.33 (96.28,97.25)	96.67 (93.66,96.71)	0.109	45.22 (33.82,218.71)	46.31 (47.70,119.31)	32.58 (33.94,149.04)	0.070
Anterior	97.83 (97.02,97.72)	97.00 (96.07,97.28)	96.00 (94.98,96.42)*	< 0.001	58.63 (70.84,152.51)	45.76 (54.53,120.04)	28.67 (37.08,56.13)*	< 0.001
Anteroseptal	98.00 (97.30,97.95)	97.50 (95.37,97.80)	97.33 (95.03,96.80)*	0.048	72.62 (67.73,289.06)	56.00 (64.29,151.62)	49.52 (63.74,186.84)	0.110
Septal	97.33 (96.53,97.38)	97.33 (96.17,97.15)	97.00 (94.21,97.05)	0.474	50.41 (23.05,252.47)	47.14 (34.78,144.99)	48.30 (56.02,110.71)	0.590

*MWI* myocardial work index, *CW* constructive work, *WW* waste work, *WE* work efficiency

*p < .05 vs normal controls

^#^p < .05 vs T2DM

## Discussion

The present study provides the followingfindings: (1) T2DM with and without HT patients have subclinical global and segmental LV myocardial systolic dysfunction, and the impairment of anterior wall is more significant. (2) TC, SBP, and MAPSE were independent influencing factors for GCW in T2DM patients. (3) The combination of GWI, GCW, GWE, and GCW/GWW was better than GWI, GCW, GWE, and GCW/GWW alone in evaluating subclinical LV myocardial systolic dysfunction in T2DM patients with and without HT (AUC = 0.743, p < 0.05).

### Myocardial work

Micro- and macrovascular lesions that cause myocardial ischaemia [17] or diabetic cardiomyopathy in the absence of dyslipidaemia, hypertension, and coronary artery disease [18] have consequences for LV myocardial systolic function in T2DM patients. However, subclinical LV myocardial systolic dysfunction is not well reflected in conventional echocardiographic parameters (LVEF is maintained within normal values). Therefore, several earlier reports proposed diastolic dysfunction as the earliest functional change during the development of myocardial dysfunction in diabetes [19, 20]. Subsequently, Ernande et al.[21] noted that there were various degrees of abnormality in LV myocardial systolic function in T2DM patients with normal LV diastolic function at early stages, as reflected by impaired strain. However, the measurement of strain was overdependent on afterload, and Russell et al. [10] proposed to estimate MW by constructing PSL from non-invasive measurement of LV peak systolic pressure to accurately evaluate LV myocardial systolic function.

MW as a novel technique has been used in recent studies to evaluate the effects of cardiovascular disease on myocardial systolic function. Edwards et al. [22] found that GWI (AUC = 0.786) was more sensitive than GLS (AUC = 0.693) in coronary artery disease patients with normal LVEF and no segmental motion abnormalities. Hiemstra et al. [23] stated that GWI, GCW and GWE were significantly decreased in non-hypertrophic cardiomyopathy patients, but GWW was increased. The prognosis of patients with GCW > 1730 mmHg% was significantly better than that of patients with GCW < 1730 mmHg%. Ilardi et al. [24] retrospectively reviewed 170 patients with moderate to severe aortic stenosis with normal LVEF and found that GWI < 1951 mmHg% or GCW < 2475 mmHg% was predictive of all-cause mortality in this population. Tadic et al. [25] studied 110 HT patients and found that GWI and GCW were significantly increased in those patients compared with normal controls. In our previous study [11], we used MW to evaluate LV myocardial systolic function in T2DM patients with preserved LVEF, and we concluded that GWI, GCW and GWE were significantly lower in T2DM patients than in normal controls and proposed that MW estimation could be used as a novel technique to evaluate subclinical LV myocardial dysfunction in T2DM patients.

Therefore, in the present study, after applying MW estimation to evaluate LV myocardial systolic function in T2DM patients with and without HT, we found that GWI, GCW, GWE, and GCW/GWW were significantly impaired compared with those in normal controls. In addition, from the normal controls to T2DM patients with HT, GWE and GCW/GWW showed a decreasing trend, and GWW showed an increasing trend. Myocardial work efficiency is derived indirectly from the ratio of constructive and wasted myocardial work [26]. GCW/GWW is an intuitive expression of work efficiency, which can provide more reference values for the increase and decrease of GCW and GWW. This finding suggests that HT has additional influencing factors on the LV myocardial systolic function of T2DM patients and causes more impairment in LV myocardial systolic function. The myocardial systolic dysfunction is closely related to the compensatory hypertrophy of LV myocytes and the proliferation of fibroblasts caused by the long-term afterload increase in HT patients and eventually leads to myocardial fibrosis. However, the upwards trend of GWI and GCW in T2DM patients with HT compared with T2DM patients has been attributed to the need for the LV myocardium to use systolic reserve to overcome the increased afterload resulting from HT, thereby maintaining LVEF within normal values. Hyperinsulinemia and insulin resistance can cause alterations in vascular homeostasis, decreases in nitric oxide and increases in reactive oxygen species levels, leading to myocardial dysfunction, and together with aberrant activation or immune dysregulation of the renin–angiotensin–aldosterone system, they promote interstitial fibrosis in cardiac tissue [27], providing an explanation for subclinical LV myocardial systolic dysfunction in T2DM patients.

Additionally, in the present study, we evaluated the segmental differences in MW in T2DM patients and T2DM patients with HT compared with normal controls to identify the negative effects of myocardial segments in T2DM patients with and without HT. The impairment of anterior wall was more evident, and the lateral, inferior, posterior, and septum were also impaired to various degrees, but no significant impairment was observed in the anteroseptal wall. Segmental myocardial systolic dysfunction may be related to abnormal lipid metabolism in T2DM patients [28]. On the one hand, with increased delivery of fatty acids to cardiomyocytes, enhanced fatty acid β-oxidation leads to myocardial hypoxia. On the other hand, an increase in lipids and their metabolites increases the risk of coronary atherosclerosis, leading to a decrease in myocardial blood supply. However, existing studies do not better explain why the impairment of the anterior myocardium is more pronounced in T2DM patients. It seems to be related to myocyte fibrosis in the corresponding segment or ischemia of the diagonal branches in the left anterior descending coronary artery. The impairment of the anterior myocardium seems to serve as reference information to suggest the formation of corresponding coronary artery lesions or regional myocardial fibrosis. Further research is needed to demonstrate its reliability for clinical guidance. On the other hand, it is not possible to exclude the possible role exerted by the chest wall conformation in determining the impairment of myocardial strain and MW parameters, particularly at basal level, in T2DM patients with a narrow antero-posterior chest diameter [29, 30].

### Clinical implications

Global and segmental analysis of MW in T2DM patients with and without HT can be more convenient and rapid for helping clinicians identify patients with global and segmental myocardial systolic dysfunction at subclinical stages with preserved LVEF. Therefore, treatment can be administered at an early stage, leading to fewer cardiovascular complications. We incorporated GCW/GWW, a new evaluation parameter, based on the original GWI, GCW, GWW and GWE. Thus, a more intuitive evaluation constructs a gain/loss relationship between work and waste. TC was independent influencing factor for the development of impaired GCW in T2DM patients with and without HT, suggesting that T2DM patients need control blood lipids to reduce the toxic effects of lipids on cardiomyocytes, also T2DM patients should control their blood pressure. These findings could be used to derive additional protocols and provide value when MW evaluation is applied to other cardiovascular diseases. In addition, it was concluded in ROC curve analysis that evaluation with a combination of the above four indices of subclinical LV myocardial systolic dysfunction was significantly better than that of single index evaluation.

## Conclusion

MW can non-invasively evaluate subclinical global and segmental LV myocardial systolic dysfunction in T2DM patients with and without HT. TC and SBP were independent influencing factors for GCW in T2DM patients with and without HT. Regulating total cholesterol levels and controlling blood pressure in T2DM patients with and without HT might reduce the impairment of LV myocardial systolic function.

## Limitations

There are still some limitations in the present study. First, patients with poor image quality due to obesity, smoking, etc., were excluded, so this exclusion criterion may be biased for different operators. Furthermore, the present study was a single-centre study, and multicentre large sample studies are needed to further provide reference value for this finding.

## Data Availability

The datasets used and analysed during the current study are available from the corresponding author on reasonable request.

## Abbreviations

BMI: Body mass index
BSA: Body surface area
SBP: Systolic blood pressure
DBP: Diastolic blood pressure
HbA1c: Glycosylated hemoglobin A1c
TC: Total cholesterol
TG: Triglyceride
HDL-C: High-density lipoprotein cholesterol
LDL-C: Low-density lipoprotein cholesterol
FPG: Fasting plasma glucose
BUN: Blood urea nitrogen
SCR: Serum creatinine
LA: Left atrial
LAV: Left atrial volume
IVS: Interventricular septum
PW: Posterior wall
LV: Left ventricular
LVEDV: LV end-diastolic volume
LVESV: LV end-systolic volume
LVEF: Left ventricular ejection fraction
MAPSE: Mitral annular plane systolic excursion
LVGLS: LV global longitudinal strain
GWI: Global myocardial work index
GCW: Global constructive work
GWW: Global waste work
GWE: Global work efficiency
